# Diseases from the Influence of the Passions

**Published:** 1884-10

**Authors:** B. W. Richardson


### DISEASES FROM THE INFLUENCE OF THE PASSIONS.

The passions which act most severely on the physical life are anger, fear, hatred, and grief. The other passions are comparatively innocuous. What is called the passion of love is not injurious until it lapses into grief and anxiety; on the contrary, it sustains the physical power. What is called ambition is of itself harmless; for ambition, when it exists purely, is a nobility, lifting its owner entirely from himself into the exalted service of mankind. It injures when it is debased by its meaner ally, pride; or when, stimulating a man to too strenuous efforts after some great object, it leads him to the performance of excessive mental or physical labor, and to the consequences that follow such efforts.

The passion called avarice, according to my experience, tends rather to the preservation of the body than to its deterioration. The avaricious man, who seems to the luxurious world to be debarring himself from all the pleasures of the world, and even to be exposing himself to the pangs of poverty, is generally placing himself in the precise conditions favorable to a long and healthy existence. By his economy, he is being saved from all the worry incident to penury; by his caution, he is being screened from all the risks incident to speculation or the attempt to amass wealth by hazardous means; by his regularity of hours and perfect appropriation of the sunlight, in preference to artificial illumination, he rests and works in periods that precisely accord with the periodicity of nature; by his abstemiousness in living, he takes just enough to live, which is precisely the right thing to do, according to the rigid natural law. Thus, in almost every particular, he goes on his way freer than other men from the external causes of all the induced diseases, and better protected than most men from the worst consequences of those diseases which spring from causes that are uncontrollable.

Effects of Anger: Of the passions I have enumerated as most detrimental to life, anger stands first. He is a man very rich indeed in physical power who can afford to be angry. The richest cannot afford it many times without insuring the penalty, a penalty that is always severe. What is still worse of this passion is, that the very disease it engenders feeds it, so that if the impulse go many times unchecked it becomes the master of the man.

The effects of passion are brought out entirely through disturbance in the organic nervous chain. We say a man was "red" with rage, or we say he was "white" with rage, by which terms, as by degrees of comparison, we express the extent of his fury. Physiologically, we are then speaking of the nervous condition of the minute circulation of his blood: that "red" rage means partial paralysis of minute blood-vessels; that "white" rage means temporary suspension of the action of the prime mover of the circulation itself. But such disturbances cannot often be produced without the occurrence of permanent organic evils of the vital organs, especially of the heart and of the brain.

The effect of rage upon the heart is to induce a permanently perverted motion, and particularly that perverted motion called intermittency, of the nature of which the reader has doubtless already been informed.

One striking example, among others of this kind which I could name, was afforded me in the case of a member of my own profession. This gentleman told me that an original irritability of temper was permitted, by want of due control, to pass into a disposition of almost persistent or chronic anger, so that every trifle in his way was a cause of unwarrantable irritation. Sometimes his anger was so vehement that all about him were alarmed for him even more than for themselves, and when the attack was over there were hours of sorrow and regret, in private, which were as exhausting as the previous rage. In the midst of one of these outbreaks of short, severe madness, he suddenly felt, to use his own expression, as if his "heart were lost." He reeled under the impression, was nauseated and faint; then, recovering, he put his hand to his wrist, and discovered an intermittent action of his heart as the cause of his faintness. He never completely rallied from that shock, and to the day of his death, ten years later, he was never free from the intermittency.

The effect of anger upon the brain is to produce first a paralysis, and afterward, during reaction, a congestion of the vessels of that organ; for, if life continues, reactive congestion follows paralysis as certainly as day follows night. Thus, in men who give way to violent rage there comes on, during the acute period, what to them is merely a faintness, which, after a time of apparent recovery, is followed by a slight confusion, a giddiness, a weight in the head, a sense of oppression, and a return to equilibrium. They are happy who, continuing their course, suffer no more severely. Many die in one or other of the two stages I have named. They die in the moment of white rage, when the cerebral vessels and heart are paralyzed. Then we say they die of faintness, during excitement. Or, they die more slowly when the rage has passed and the congestion of reaction has led to engorgement of the vessels of the brain. Then the engorgement has caused stoppage of the circulation there; or a vessel has given way; or serous fluid has exuded, producing pressure, and we report that the death was apoplexy, following upon excitement.

EFFECTS OF HATRED: Hatred, when it is greatly intensified, acts much like anger in the effects it produces. The phenomena differ in that they are less suddenly developed and more closely concealed—they very rarely, in fact, come under the cognizance of the physician unmixed with other phenomena. They are made up of the symptoms of suppressed anger with morose determination, and they keep the sufferer from rest. He is led to neglect the necessities of his own existence; he is rendered feverish and feeble; and at last he either sinks into chronic despondency and irritability, or rushes hastily to the performance of some act which indicates disordered mind.

EFFECTS OF FEAR: The effects of fear are all but identical with those of rage, and, like rage, grow in force with repetition. The phenomena are so easily developed in the majority of persons that they may actually be acquired by imitation, and may be intensified and perhaps induced by listening to the mere narratives of events which act as causes of fear. I am daily more and more convinced that not half the evils resulting from what may be called the promptings of fear in the young and the feeble are duly appreciated, and that fear is the worst weapon of physical torture the thoughtless coward wields. The organs upon which fear exerts its injurious influence are, again, the organic nervous chain, the heart, and the brain.

Permanent intermittency of the heart is one of the leading phenomena incident to sudden and extreme terror. One example, sufficiently characteristic, will illustrate this fact.

A gentleman of middle age was returning home from a long voyage in the most perfect health and spirits, when the vessel in which he was sailing was struck from a collision, and, hopelessly injured, began to sink. With the sensation of the sinking of the ship and the obvious imminence of death—five minutes was the longest expected period of remaining life—this gentleman felt his heart, previously acting vehemently, stop in its beat. He remembered then a confused period of noise and cries and rush, and a return to comparative quiet, during which he discovered himself being conveyed, almost unconsciously, out of the sinking vessel on to the deck of another vessel that had rendered assistance. When he had gained sufficient calmness he found that periods of intermittent action of his heart could be counted. They occurred four and five times in the minute for several days, and interfered with his going to sleep for many nights.

The effect of fear on the brain may be to the extent of that which is produced by extremity of rage, so that even sudden death, from syncope, may ensue. I have known two such instances as these; but the more common effect is an intense irritability, followed by doubt, suspicion, and distrust, leading toward or to insanity. From a sudden terror deeply felt the young mind rarely recovers, never, I believe, if hereditary tendency to insanity be a part of its nature. A man who is now the inmate of an asylum, owing to fixed delusions that all his best friends are conspiring to injure and kill him, explained to me, before his delusion was established, from what it started. When he was a boy he had a nervous dread of water, and his father, for that very reason and with the best of intentions, determined that he should be taught to swim. He was taken by his tutor, in whom he had every confidence, to the side of a river, and when he was undressed he suddenly found himself cast by his instructor, without any warning, into the stream. No actual danger of drowning was implied, for the tutor himself was at once in the water to hold him up or to bring him to land; but the immediate effect, beginning with the faintness of fear, was followed by vomiting, by a long train of other nervous symptoms, by constant dread that some one was in some way about to repeat the infliction, by frequent dreaming of the event by night, by thinking upon it in the day. At last, all the phenomena culminated in that breach between the instinctive and the reasoning powers which we, for want of a better term, call dangerous and insane delusion.

EFFECTS OF GRIEF: The effect of grief varies somewhat according to the suddenness or slowness with which it is expressed. Sudden grief tells chiefly upon the heart, leading to irregular action and to various changes in the extreme parts of the circulation incidental to such irregularity. Under sudden impulse of grief I have known singular local manifestations of disease, as, for instance, the development of a *goitre;* an hæmoptysis or loss of blood from the lungs; a local paralysis of the lip and tongue; a failure of sight.

When the grief is less sudden and more prolonged, want of power and intermittency of the circulation are again the most common phenomena. They are most easily developed in women, but I have seen them occur even in men of strong habit but sensitive feeling. Thus a gentleman whom I know well, and who suffers in the way I describe, tells me that he first became conscious of the intermittency in the action of his heart, upon the anxiety he felt from the loss of one of his brothers to whom he was deeply attached, and for whose superior talents he had, as indeed many others had, a profound admiration. The attacks at first were so severe that they created in his mind some alarm; but in course of time he became accustomed to them and the sense of fear passed away. The intermittency in this instance alternated with periods in which there was very slight interruption of natural action. During the more natural periods there was, however, an occasional absence of stroke once in two or three hundred beats, but the fact was not evident to the subject himself. When the extreme attacks were present the intermittency of pulse occurred six or even seven times in the minute, and the fact, which was subjectively felt, was very painful. The stomach at the same time was uneasy; there was flatulency and a sensation of sinking and exhaustion. In the worst attacks there was also some difficulty in respiration and a desire for more capacity for air, but unattended by spasm or acute pain. A severe attack was induced readily by any cause of disturbance, such as broken rest or mental excitement; on the other hand, rest and freedom from care seemed to him curative for a time.

In this gentleman another symptom was presented for one or two years which is somewhat novel and exceedingly striking. The symptom was this. When the intermittent action of the heart was at its worst, there came on in the fingers of one or other hand a sensation of coldness and numbness, followed instantly by quick blanching of the skin, precisely the same appearance, in fact, as is produced when the surface of the body is frozen. The numbness and temporary death of the parts would often remain for a full hour, during which time the superficial sensibility was altogether lost. When recovery commenced in the fingers it was very rapid, and after recovery no bad results were ever noticeable. I have since seen one similar illustration in another individual, occurring under nearly similar circumstances.

From the irregularity of the circulation of the blood induced by prolonged grief, varied central phenomena in the nervous matter follow, and in persons who have passed middle life these phenomena are usually permanent if not progressive. They consist of organic feebleness extending to all the active organs of the body, and affecting specially the mental organism. A constant desire for rest, for avoidance of cares, for seclusion, mark this stage of disease, if so it may be called. It is not necessarily a stage leading to rapid failure of further physical or mental power, for the mind and body are subdued so equally there is no galling irritability, no wearing depression from the influence of other passions. The worst that happens ultimately in those instances is the gradual but premature encroachment of dementia previous to death, if the life be prolonged to its natural term.—Dr. B. W. Richardson.

